# Supplementary material for: Hydrogenosomal tail-anchored proteins are targeted to both mitochondria and ER upon their expression in yeast cells
Source: PLoS One. 2020 Aug 20;15(8):e0237982. doi: 10.1371/journal.pone.0237982 (PMC7446849; doi:10.1371/journal.pone.0237982)
Supplement: S1 Raw images — (PDF) [file pone.0237982.s001.pdf]

Original Blots

Fig. 1A

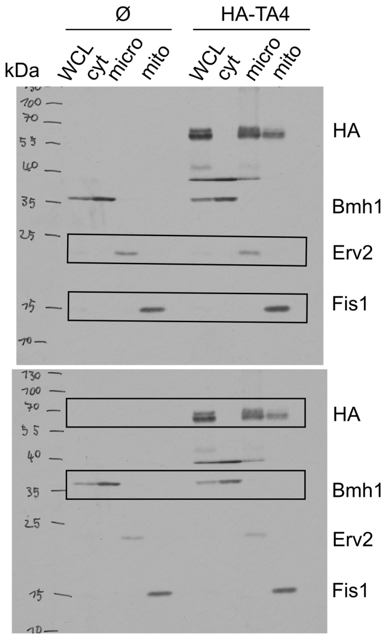

Fig. 1B

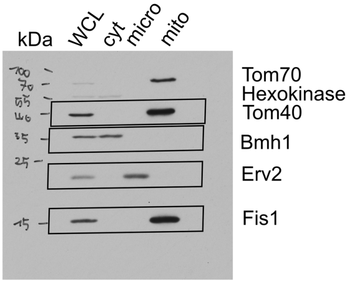

Fig. 1D

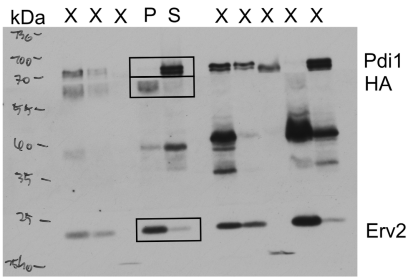

Fig. 1E

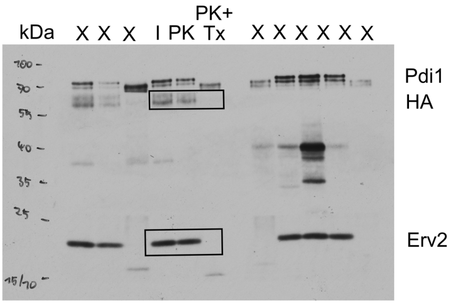
kDa
X X X I PK PK+ Tx X X X X X X
100
70
55
40
35
25
15/10
Pdi1
HA
Erv2

Fig. 1F

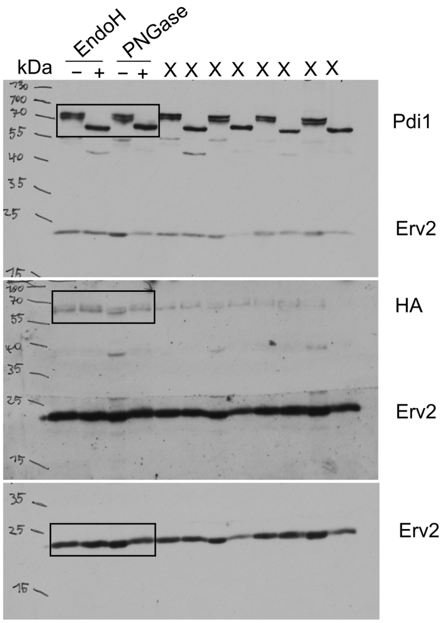
EndoH
PNGase
kDa
− + − + X X X X X X X X
130
100
70
55
40
35
25
15
Pdi1
Erv2
HA
Erv2
Erv2

Fig. 1G

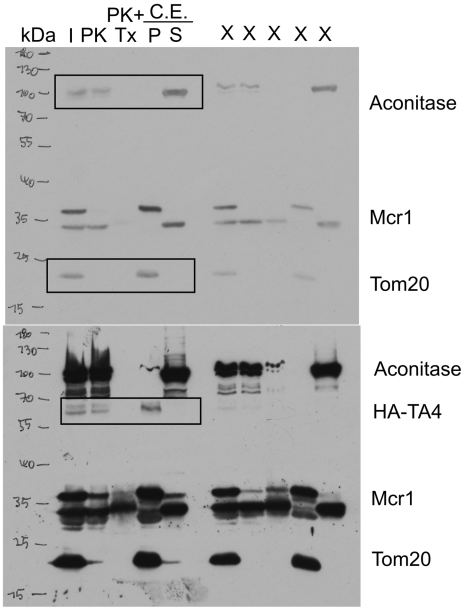
PK+
C.E.
kDa
I
PK
Tx
P
S
X X X X X
Aconitase
Mcr1
Tom20
Aconitase
HA-TA4
Mcr1
Tom20

Fig. 1H

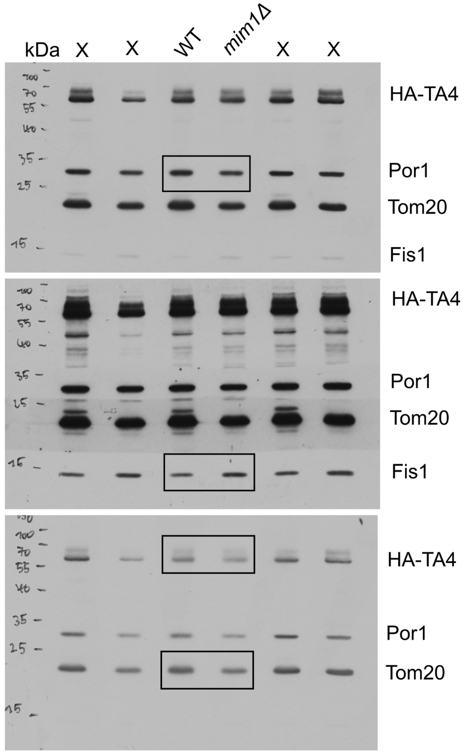
kDa
X
X
WT
mim1Δ
X
X
HA-TA4
Por1
Tom20
Fis1
HA-TA4
Por1
Tom20
Fis1
HA-TA4
Por1
Tom20

Fig. 2A

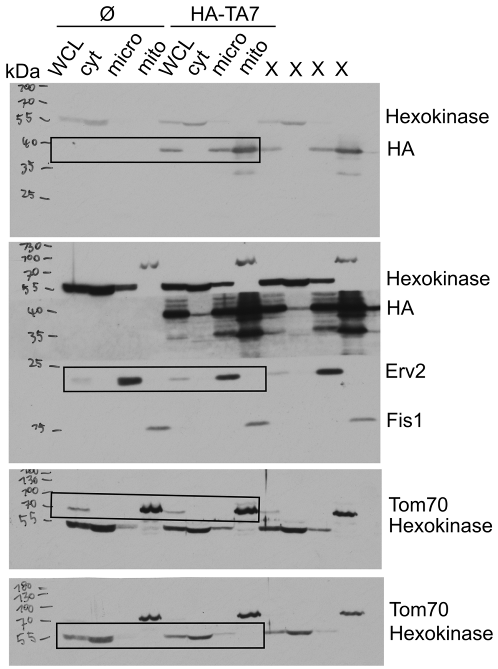

Ø
HA-TA7
kDa
WCL
cyt
micro
mito
WCL
cyt
micro
mito
X X X X
Hexokinase
HA
Hexokinase
HA
Erv2
Fis1
Tom70
Hexokinase
Tom70
Hexokinase

Fig. 2C

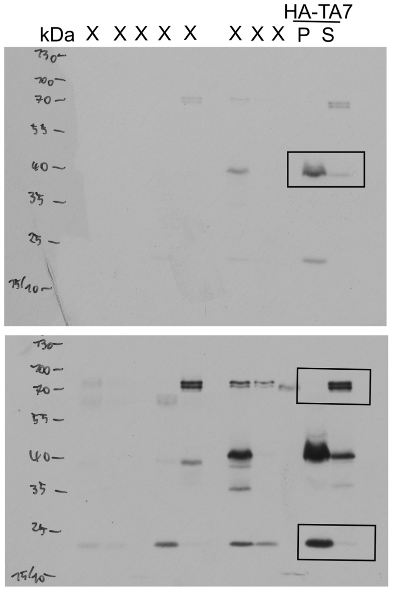

HA-TA7
kDa
X X X X X X X X P S
Pdi1
HA
Erv2
Pdi1
HA
Erv2

Fig. 2D

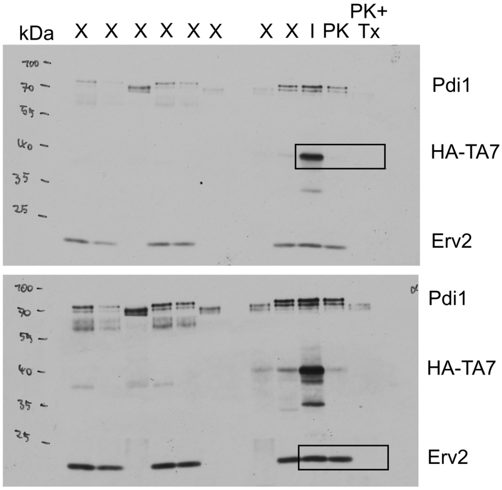
kDa
X X X X X X X X I PK PK+ Tx
Pdi1
HA-TA7
Erv2
Pdi1
HA-TA7
Erv2

Fig. 2E

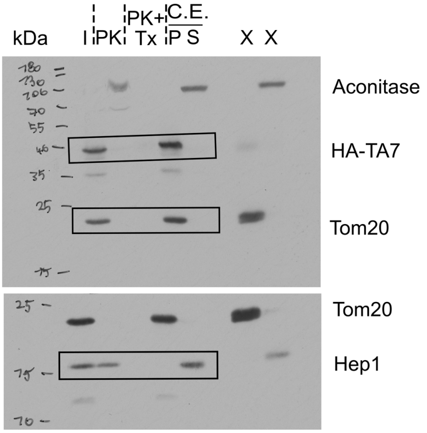
kDa
I PK PK+ Tx C.E. P S X X
Aconitase
HA-TA7
Tom20
Tom20
Hep1

Fig. 2F

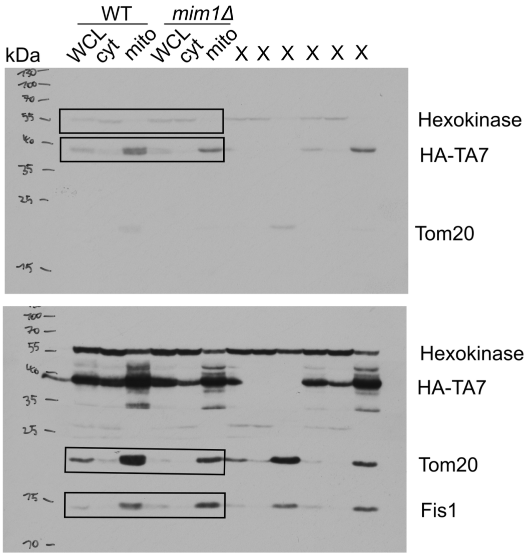

Fig. 3A

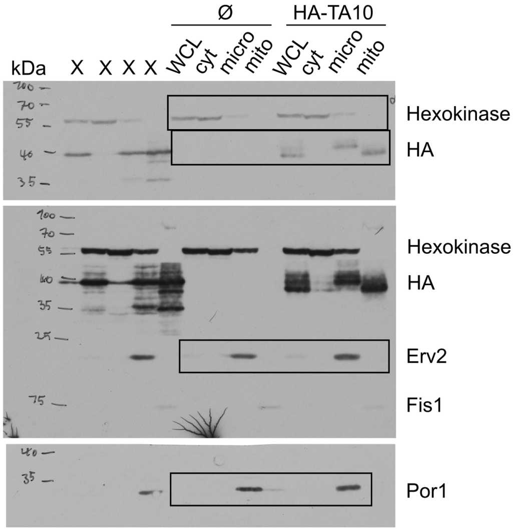

Fig. 3C

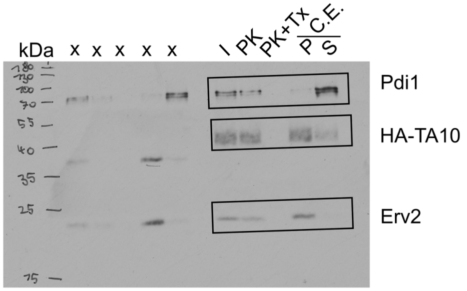

Fig. 3D

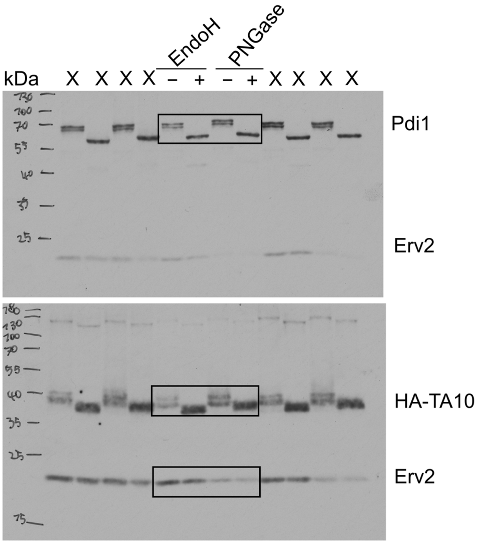
EndoH
PNGase
kDa
X X X X – + – + X X X X
Pdi1
Erv2
HA-TA10
Erv2

Fig. 3E

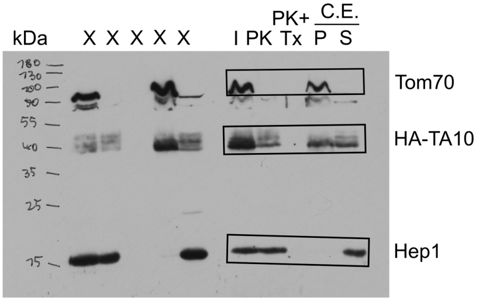
PK+
C.E.
kDa
X X X X X
I PK Tx P S
Tom70
HA-TA10
Hep1

Fig. 3F

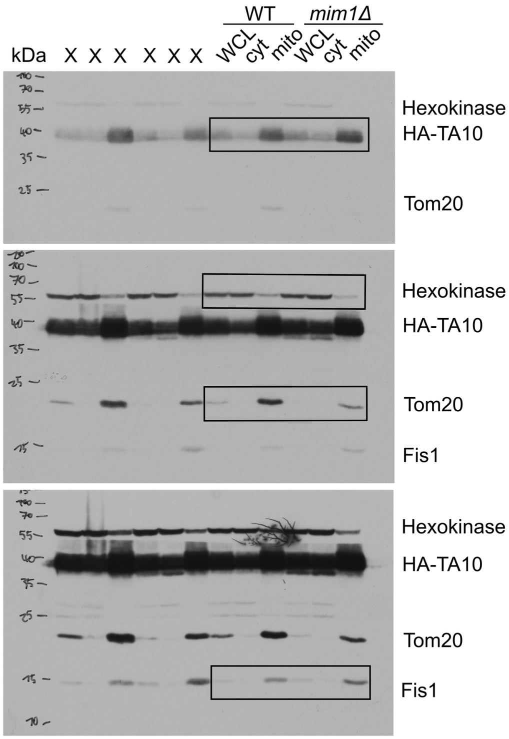

WT
mim1Δ
kDa
X X X X X X
WCL cyt mito WCL cyt mito
Hexokinase
HA-TA10
Tom20
Fis1

Fig. 4B

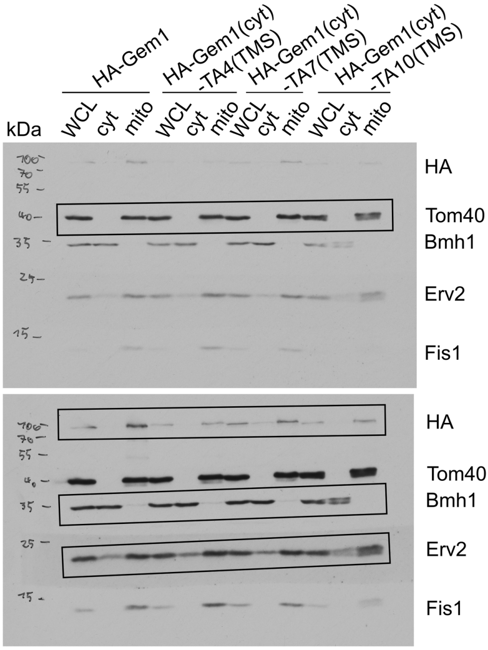

HA-Gem1
HA-Gem1(cyt) -TA4(TMS)
HA-Gem1(cyt) -TA7(TMS)
HA-Gem1(cyt) -TA10(TMS)
kDa
WCL cyt mito WCL cyt mito WCL cyt mito WCL cyt mito
HA
Tom40
Bmh1
Erv2
Fis1
