# Supplementary material for: Hydrogenosomal tail-anchored proteins are targeted to both mitochondria and ER upon their expression in yeast cells
Source: PLoS One. 2020 Aug 20;15(8):e0237982. doi: 10.1371/journal.pone.0237982 (PMC7446849; doi:10.1371/journal.pone.0237982)
Supplement: Attachment — Submitted filename: Ferdigg et al. - Response to reviewers.docx [file pone.0237982.s003.docx]

**Addressing the comments of the reviewers**

***Reviewer #2:***

We thank the reviewer for his/her generally positive opinion on our contribution.

*The revised version of the manuscript PONE-D-20-08560R1 has improved substantially and the reviewer requests were generally properly fulfilled. There is just one last point of criticism regarding a conclusions of this study. On page 26, line 529-531 the authors conclude that their "study suggests that TA proteins might have evolved after mitochondria became hydrogenosomes". With regard to the results presented, such an assumption is not justified. As the authors cite in the manuscript, also mammalian mitochondrial TA-proteins have been found to mistarget to the ER in the same fashion as the Trichomonas TA proteins investigated. Thus, also mammals have developed a TA targeting system which differs substantially from the yeast system. However, they are evolutionary closer related to yeast than Trichomonas and contain normal mitochondria. In the light of these discrepancies the sentence should be removed from the manuscript.*

The reviewer raised a valid point and we agree that the conclusion that “TA proteins might have evolved after mitochondria became hydrogenosomes” is merely a speculation. Therefore, we removed that sentence from the revised manuscript.
